# A Case of Philadelphia Chromosome-Positive Acute Lymphoblastic Leukemia in a 26-Year-Old Pregnant Woman

**DOI:** 10.7759/cureus.60679

**Published:** 2024-05-20

**Authors:** Natalie Shaykh, Falguni Patel, Luke Stachler, Kabeer Ali, Vanshika Tripathi, Oshin Rai, Rafik Jacob

**Affiliations:** 1 Internal Medicine, University of Florida College of Medicine – Jacksonville, Jacksonville, USA

**Keywords:** chemotherapy, tki therapy, imatinib therapy, cancer in pregnancy, bcr-abl positive, philadelphia chromosome positive acute lymphoblastic leukemia (ph+ all)

## Abstract

Acute lymphoblastic leukemia (ALL) is an uncommon and rapidly progressing blood cancer originating in the bone marrow, characterized by the abnormal proliferation of immature lymphocytes. Although most cases of ALL are observed in children, the disease pattern shows two peaks: one in early childhood and another around the age of 50. Approximately a fifth to a third of adults diagnosed with ALL exhibit cytogenetic abnormalities involving the Philadelphia chromosome. Despite the existence of several studies on Philadelphia chromosome-positive ALL (Ph+ ALL), our case accentuates the use of a multi-disciplinary approach to treatment and involves a patient from a unique demographic.

## Introduction

Acute lymphoblastic leukemia (ALL) is an aggressive hematological malignancy consisting of blastic transformation of the T or B cell lineages [1]. While the disease has been more commonly associated with the pediatric population, the incidence is bimodal, with peaks around three years old and greater than 50 years old. It is the predominant leukemia in the pediatric population, comprising up to 80% of cases in this group compared to 20% in adults [1]. Clinical signs at presentation include constitutional symptoms alongside manifestations like easy bruising, bleeding, arthralgias, dyspnea, and infections [2]. The largest genetically defined subtype of adult ALL is Philadelphia chromosome-positive ALL (Ph+ ALL), which used to have an unfavorable prognosis until recent treatment advancements [3]. Tyrosine kinase inhibitors (TKIs), such as imatinib, emerged as a fundamental component of initial therapy for Ph+ ALL, achieving remission rates of over 90% regardless of whether imatinib is administered as monotherapy or in conjunction with chemotherapy [3]. Delays in managing acute leukemia can negatively impact the mother’s prognosis, yet administering chemotherapy during pregnancy can lead to severe adverse effects on the fetus [4]. Herein is a case report of a young pregnant patient diagnosed with ALL outlining the complexities of treatment.

## Case presentation

**Figure 1:**
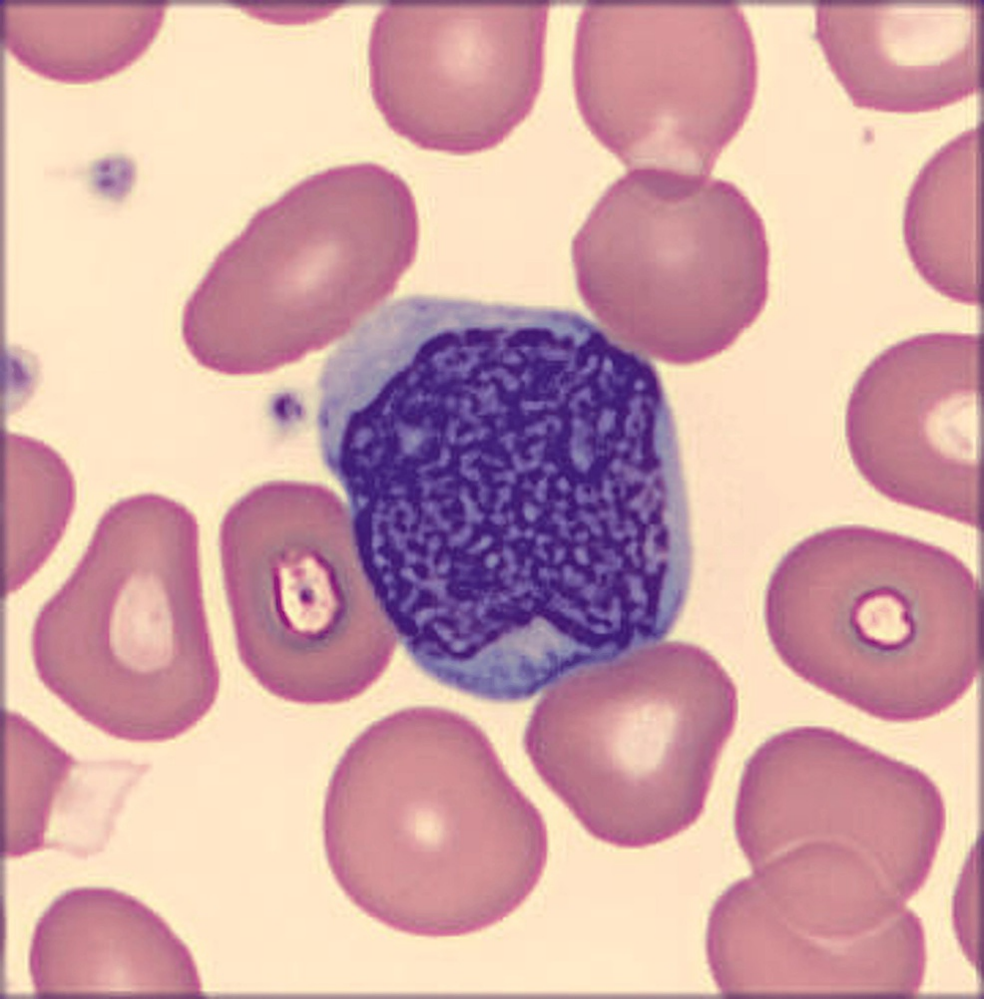
Blast cell on peripheral smear Figure 1 demonstrates the patient's peripheral smear revealing a typical blast cell - an immature white blood cell often larger than other cells and characterized by a high nucleus-to-cytoplasm ratio. These cells are typically uncommon or found in minimal quantities on a peripheral smear.

A 26-year-old female, 29 weeks and six days pregnant with a past medical history of Hashimoto’s disease and severe persistent asthma, was referred to Hematology Oncology in October 2023 for anemia, leukocytosis, and thrombocytosis, for which she was taking daily aspirin. The patient’s only complaint at this time was fatigue, which she attributed to her pregnancy. She denied any fever, chills, lymph node enlargement, weight loss, night sweats, early satiety, infections, or rashes. She reported poor tolerance to oral iron but never required intravenous (IV) iron in the past. The complete blood count with differential seen in Table 1 was significant for 49,950 white blood cells/µL, hemoglobin of 10.6 g/dL, and 825,000 platelets/µL with differential notable for 4% metamyelocytes, 5% myelocytes, and 13% blasts, as demonstrated in Figure 1.

**Table 1:** Complete blood count with differential The patient's complete blood count with differential on initial presentation. MCH, mean corpuscular hemoglobin; MCHC, mean corpuscular hemoglobin concentration; MCV, mean corpuscular volume; MPV, mean platelet volume; RDW, red cell distribution width

Complete blood count	Result	Reference range
WBC	49.95	4.0-10.0 × 10^3^/µL
RBC	3.99	4.0-5/2 × 10^6^/µL
Hemoglobin	10.6	12.0-16.0 g/dL
Hematocrit	32.4	35.0-45.0%
MCV	81.2	78.0-100.0 fL
MCH	26.6	26.0-34.0 pg
MCHC	32.7	31.0-36.0 g/dL
RDW	14.5	11.0-14.6%
Platelet count	825	150-450 × 10^3^/µL
MPV	9.5	9.5-12.2 fL
Neutrophils %	40	40.0-80.0%
Bands %	5	0-10%
Lymphocytes %	13	20.0-45.0%
Monocytes %	3	2.0-10.0%
Eosinophils %	6	0.0-8.0%
Nucleated RBC %	0.4	0.0-1.0%
Basophils %	6	0.0-2.0%
Metamyelocytes	4	≤0%
Myelocytes	5	≤0%
Promyelocytes	0	≤0%
Blasts %	13	≤0%
Lymphocytes absolute	9.14	1.0-3.2 × 10^3^/µL
Eosinophils absolute	2.90	0.03-0.46 × 10^3^/µL
Basophils absolute	2.90	0.02-0.09 × 10^3^/µL
Immature granulocytes absolute	4.60	≤0.0 × 10^3^/µL
Monocytes absolute	1.65	0.2-0.9 × 10^3^/µL
Neutrophils absolute	22.53	1.4-7.5 × 10^3^/µL
Atypical lymphocytes %	5.8	0-10%

Flow cytometry from peripheral blood showed 25% lymphoblasts, a cluster of differentiation (CD) 19, CD34, CD10, terminal deoxynucleotidyl transferase (TdT), and partial CD13 prompting the decision for inpatient admission. Bone marrow biopsy was completed, and the patient received 40 mg of IV dexamethasone, as it acts as an important part of treatment in those with ALL. After extensive discussion with the obstetrics-gynecology team, the patient underwent a successful cesarean section and was continued on IV steroids with gastrointestinal prophylaxis in preparation for chemotherapy. In the meantime, echocardiography showed normal cardiac function. The results of the bone marrow biopsy returned showing hypercellular marrow with 24% B lymphocytes along with positive fluorescent in situ hybridization (FISH) studies for the breakpoint cluster region-Abelson murine leukemia viral oncogene homolog 1 (BCR-ABL1) gene, suggestive of Ph+ ALL. The patient was started on allopurinol and IV fluids. A lumbar puncture showed infiltrated cerebrospinal fluid (CSF) with 15% CD19/CD10 positive lymphoblasts but numerous red blood cells, so contamination could not be excluded. Subsequently, it was decided to start the patient on hyperfractionated Cyclophosphamide, Vincristine, Doxorubicin, and Dexamethasone (Hyper-CVAD) with imatinib. Given possible CSF infiltration in the diagnostic lumbar puncture, the patient started with cycle B of Hyper-CVAD, including Methotrexate and Cytarabine. Over the next several months, the patient completed five cycles of this treatment, with her most recent biopsy marrow biopsy in February 2024 showing minimal residual disease. She is to be seen for pre-evaluation at a transplant facility prior to proceeding with a hematopoietic stem cell transplantation (HSCT).

## Discussion

In general, age is an important factor in determining the prognosis of ALL, with worse outcomes as the age at diagnosis increases. In our patient, being 26 years old confers a long-term survival of only 50% to 60% versus 80% in children less than five years old and 30% in adults 45 to 54. [5] Poor outcomes in older patients are thought to be due to more unfavorable cytogenetic abnormalities. The most common one, as seen in this case, is that of the Philadelphia (Ph) chromosome, a translocation between chromosomes 9 and 22 that creates a fusion gene called BCR-ABL. This fusion gene produces a protein with enhanced tyrosine kinase activity, leading to uncontrolled cell growth and proliferation.

Historically, only HSCT provided the chance of cure [3]. TKIs, however, quickly became a pivotal component of treatment, addressing the critical need for effective non-transplant therapy with the potential for cure, thereby bridging the gap before allogeneic HSCT [6]. The standard initial treatment for Ph+ ALL now typically involves imatinib combined with chemotherapy, as responses to imatinib monotherapy tend to be short-lived and resistance to treatment develops rapidly [5]. Other studies have looked at the effect of second-generation TKIs as front-line treatment for Ph+ ALL, and the results were promising, showing high molecular response rates [3].

While cancer in pregnancy is relatively rare, breast cancer and cervical cancer are among the most prevalent tumors detected, followed by melanoma, leukemia, and lymphoma [7]. For pregnant patients, careful attention should be paid to the chemotherapy regimen, including dosages, as well as to the gestational age of the fetus during chemotherapy administration to minimize fetal exposure while ensuring that the mother receives optimal therapy [7]. Moreover, treating leukemia during pregnancy poses a formidable challenge, requiring collaborative efforts from a multidisciplinary team spanning from hematologists to obstetricians [8]. Following this approach with our patient, the team opted for a cesarean section at 30 weeks as the safest course of action, which would subsequently allow prompt initiation of treatment for the patient. Although certain studies have indicated elevated risks of fetal abnormalities or spontaneous abortions associated with Imatinib use during pregnancy, our patient had already given birth to her premature, otherwise healthy baby and did not want to breastfeed [9]. Hyper-CVAD combination therapy was chosen for induction treatment due to its demonstrated effectiveness in adult ALL. Additionally, components of this regimen, including Doxorubicin, Cyclophosphamide, and Vincristine, have commonly been utilized during pregnancy, particularly in the treatment of breast cancer and lymphoma, with favorable outcomes [7].

## Conclusions

ALL is infrequently encountered during pregnancy and demonstrates a bimodal distribution, with most cases arising in early childhood and later adulthood. Maintaining a heightened awareness of malignancy during pregnancy, however, is paramount, as normal physiologic changes can mask leukemia symptoms. Early detection and a multidisciplinary approach, coupled with personalized management based on factors, such as gestational stage and cytogenetics, are crucial for tailoring treatment strategies to ensure safety for both the mother and the baby.
